# Genome sequence of the dark pink pigmented *Listia bainesii* microsymbiont *Methylobacterium* sp. WSM2598

**DOI:** 10.1186/1944-3277-9-5

**Published:** 2014-12-08

**Authors:** Julie Ardley, Rui Tian, John Howieson, Ron Yates, Lambert Bräu, James Han, Elizabeth Lobos, Marcel Huntemann, Amy Chen, Konstantinos Mavromatis, Victor Markowitz, Natalia Ivanova, Amrita Pati, Lynne Goodwin, Tanja Woyke, Nikos Kyrpides, Wayne Reeve

**Affiliations:** 1 Centre for Rhizobium Studies, Murdoch University, Murdoch, Western Australia, Australia; 2 Department of Agriculture and Food, South Perth, Western Australia, Australia; 3 School of Life and Environmental Sciences, Deakin University, Melbourne, Victoria, Australia; 4 DOE Joint Genome Institute, Walnut Creek, California, USA; 5 Biological Data Management and Technology Center, Lawrence Berkeley National Laboratory, Berkeley, California, USA; 6 Los Alamos National Laboratory, Bioscience Division, Los Alamos, New Mexico, USA; 7 Department of Biological Sciences, King Abdulaziz University, Jeddah, Saudi Arabia

**Keywords:** Root-nodule bacteria, Nitrogen fixation, Symbiotic specificity, *Alphaproteobacteria*

## Abstract

Strains of a pink-pigmented *Methylobacterium* sp. are effective nitrogen- (N_2_) fixing microsymbionts of species of the African crotalarioid genus *Listia.* Strain WSM2598 is an aerobic, motile, Gram-negative, non-spore-forming rod isolated in 2002 from a *Listia bainesii* root nodule collected at Estcourt Research Station in South Africa. Here we describe the features of *Methylobacterium* sp. WSM2598, together with information and annotation of a high-quality draft genome sequence. The 7,669,765 bp draft genome is arranged in 5 scaffolds of 83 contigs, contains 7,236 protein-coding genes and 18 RNA-only encoding genes. This rhizobial genome is one of 100 sequenced as part of the DOE Joint Genome Institute 2010 ***G*** *enomic* ***E*** *ncyclopedia* for ***B*** *acteria* and ***A*** *rchaea*-***R*** *oot* ***N*** *odule* ***B*** *acteria* (GEBA-RNB) project.

## Introduction

Nodulated legumes are important and established components of Australian agricultural systems: the value of atmospheric nitrogen (N_2_) fixed by rhizobia in symbiotic association with these legumes is estimated to be worth more than $2 billion annually [1,2]. The major agricultural region of south-western Australia has a Mediterranean climate, with soils that are often acid, have a low clay content and low organic matter, and tend to be inherently infertile [3,4]. The last forty years, however, have seen a sharp decrease in average winter rainfall by about 15–20% [5]. This, together with the development of dryland salinity [6], has challenged the sustainability of using the commonly sown subterranean clover and annual medics as pasture legumes in these systems. Alternative perennial legume species (and their associated rhizobia) are therefore being sought [2]. We have identified a suite of South African perennial, herbaceous forage legumes, including several species in the crotalarioid genus *Listia* (previously *Lotononis*) [7], that are potentially well-adapted to the arid climate and acid, infertile soils of the target agricultural areas.

*Listia* species are found in seasonally wet habitats throughout southern and tropical Africa [8]. They produce stoloniferous roots [8,9] and form lupinoid nodules rather than the indeterminate type found in other crotalarioid species [7,10]. Rhizobial infection occurs by epidermal entry rather than via root hair curling [7]. *Listia*-rhizobia symbioses are highly specific. The tropically distributed *L. angolensis* forms effective (i.e. N_2_-fixing) nodules with newly described species of *Microvirga*[11], while all other studied *Listia* species are only nodulated by strains of pigmented methylobacteria [7,10,12]. Unlike the methylotrophic *Methylobacterium nodulans*, which specifically nodulates some species of *Crotalaria*[13], the *Listia* methylobacteria are unable to utilize methanol as a sole carbon source [14]. In Australia, strains of pigmented methylobacteria have been used as commercial inoculants for *Listia bainesii* and are able to persist in acidic, sandy, infertile soils, while remaining symbiotically and serologically stable [10,15].

A pigmented *Methylobacterium* strain, WSM2598, isolated from a root nodule of *L. bainesii* cv “Miles” in South Africa in 2002, was found to be a highly effective nitrogen fixing microsymbiont of both *L. bainesii* and *Listia heterophylla* (previously *Lotononis listii*) [10]. Here we present a set of preliminary classification and general features for *Methylobacterium* sp. strain WSM2598, together with the description of the genome sequence and annotation.

## Organism information

**Figure 1:**
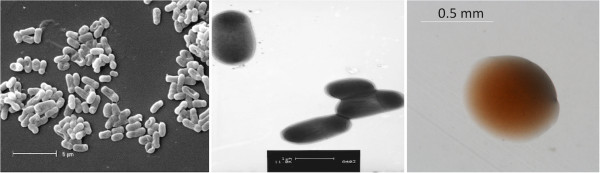
**Images of *Methylobacterium *** **sp. strain WSM2598 using scanning (Left) and transmission (Center) electron microscopy as well as light microscopy to visualize colony morphology on solid ½LA [**[10]**] (Right).**

*Methylobacterium* sp. strain WSM2598 is a motile, non-sporulating, non-encapsulated, Gram-negative rod with one to several flagella. It is a member of the family *Methylobacteriaceae* in the class *Alphaproteobacteria*. The rod-shaped form varies in size with dimensions of approximately 0.5 μm in width and 1.0-1.5 μm in length (Figure 1 Left and 1 Center). WSM2598 is medium to slow growing, forming 0.5-1.5 mm diameter colonies within 6–7 days at 28°C. WSM2598 is pigmented, an unusual property for rhizobia. When grown on half strength Lupin Agar (½LA) [10], WSM2598 forms dark pink pigmented, opaque, slightly domed colonies with smooth margins (Figure 1 Right).

WSM2598 alkalinizes ½LA containing universal indicator (BDH Laboratory Supplies). WSM2598 cultured in minimal medium [16] is unable to utilize arabinose, galactose, glucose, mannitol, methanol, methylamine or formaldehyde as sole carbon sources, but grows poorly on formate and well on succinate and glutamate [14]. Minimum Information about the Genome Sequence (MIGS) is provided in Table 1 and Additional file 1: Table S1.

**Table 1:** **Classification and general features of *Methylobacterium *** **sp. strain WSM2598 according to the MIGS recommendations **[17,18]

**MIGS ID**	**Property**	**Term**	**Evidence code**
	Current classification	Domain *Bacteria*	TAS [18]
		Phylum *Proteobacteria*	TAS [19]
		Class *Alphaproteobacteria*	TAS [20,21]
		Order *Rhizobiales*	TAS [21,22]
		Family *Methylobacteriaceae*	TAS [21,23]
		Genus *Methylobacterium*	TAS [24-26]
		Species *Methylobacterium* sp.	TAS [10]
		Strain WSM2598	TAS [10]
	Gram stain	Negative	IDA
	Cell shape	Rod	IDA
	Motility	Motile	IDA
	Sporulation	Non-sporulating	NAS
	Temperature range	Mesophile	IDA
	Optimum temperature	28°C	NAS
	Salinity	Non-halophile	NAS
MIGS-22	Oxygen requirement	Aerobic	IDA
	Carbon source	Formate, succinate & glutamate	TAS [14]
	Energy source	Chemoorganotroph	TAS [14]
MIGS-6	Habitat	Soil, root nodule on host	TAS [10]
MIGS-15	Biotic relationship	Free living, symbiotic	TAS [10]
MIGS-14	Pathogenicity	Non-pathogenic	NAS
	Biosafety level	1	TAS [27]
	Isolation	Root nodule of *Listia bainesii*	TAS [10]
MIGS-4	Geographic location	Estcourt Research Station, South Africa	TAS [10]
MIGS-5	Sample collection date	May 27, 2002	TAS [10]
MIGS-4.1	Latitude	-29.9125	TAS [10]
MIGS-4.2	Longitude	29.16667	TAS [10]
MIGS-4.3	Depth	Not reported	NAS
MIGS-4.4	Altitude	1,200 m	IDA

Evidence codes – IDA: Inferred from Direct Assay; TAS: Traceable Author Statement (i.e., a direct report exists in the literature); NAS: Non-traceable Author Statement (i.e., not directly observed for the living, isolated sample, but based on a generally accepted property for the species, or anecdotal evidence). These evidence codes are from the Gene Ontology project [31].

**Figure 2:**
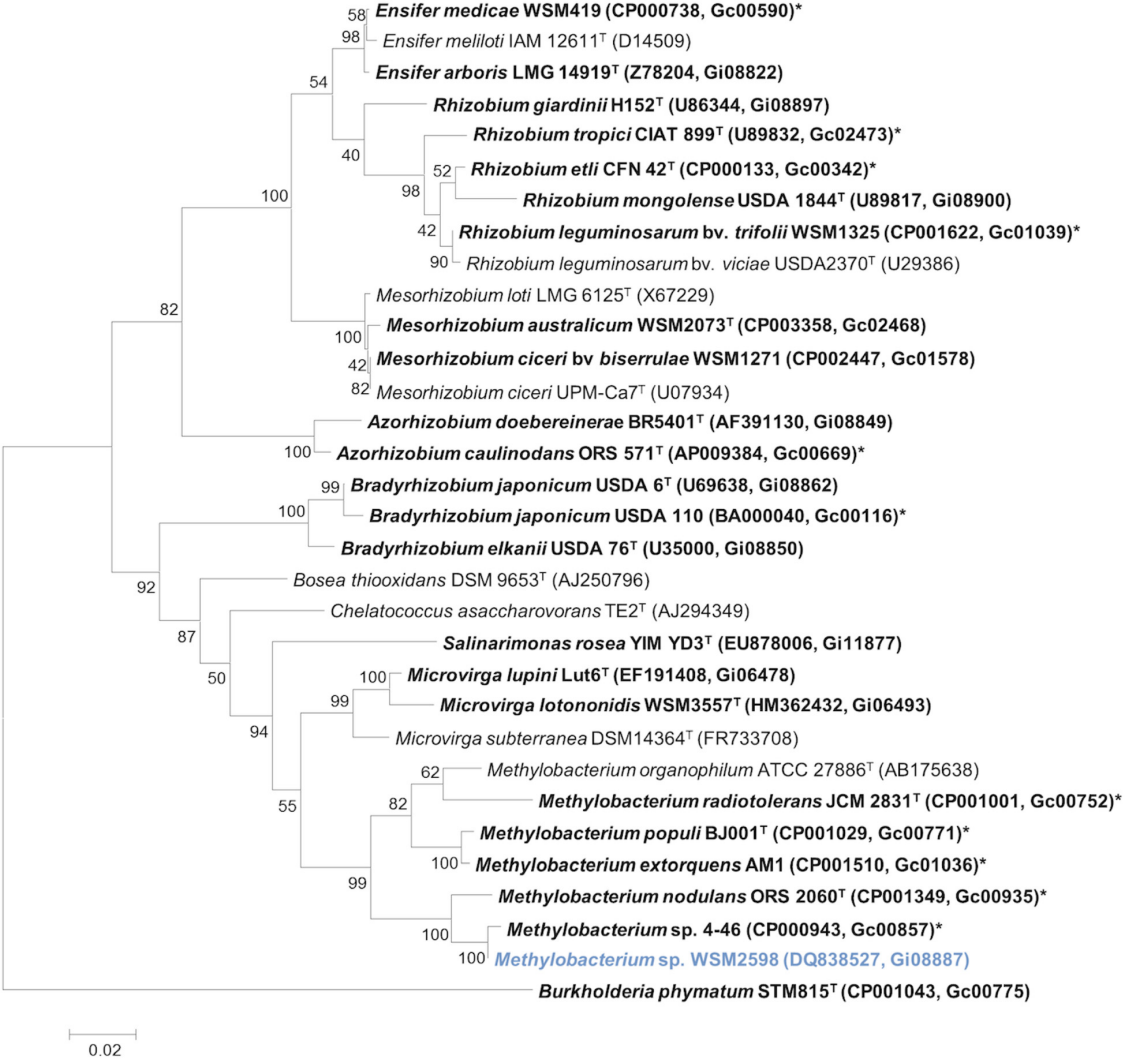
**Phylogenetic tree showing the relationships of *Methylobacterium *sp. WSM2598 (shown in blue print) with some of the root nodule bacteria in the order *Rhizobiales *based on aligned sequences of the 16S rRNA gene (1,340 bp internal region).** All sites were informative and there were no gap-containing sites. Phylogenetic analyses were performed using MEGA, version 5 [28]. The tree was built using the maximum likelihood method with the General Time Reversible model. Bootstrap analysis [29] with 500 replicates was performed to assess the support of the clusters. Type strains are indicated with a superscript T. Brackets after the strain name contain an accession number. Strains with a genome sequencing project registered in GOLD [30] are in bold print and the GOLD ID is mentioned after the accession number. Published genomes are designated with an asterisk.

Figure 2 shows the phylogenetic neighborhood of *Methylobacterium* sp. WSM2598 in a 16S rRNA sequence based tree. The 16S rDNA sequence of WSM2598 has 99% (1,358/1,364 bp) and 98% (1,334/1,365 bp) sequence identity to the 16S rRNA of the fully sequenced strains *Methylobacterium* sp. 4–46 (Gc00857) and *M. nodulans* ORS2060 (Gc00935), respectively.

### Symbiotaxonomy

*Methylobacterium* sp. WSM2598 forms nodules on (Nod^+^), and fixes N_2_ (Fix^+^), with southern African species of *Listia*. On *Listia angolensis*, some species of the crotalarioid genus *Leobordea* and the promiscuous legume *Macroptilium atropurpureum,* WSM2598 forms white, ineffective (Fix-) nodules. It does not form nodules on other tested legumes [7], [Table 2].

**Table 2:** **Compatibility of *Methylobacterium *** **sp. WSM2598 with 11 host legume genotypes for nodulation (Nod) and N**_**2**_**-Fixation (Fix)**

**Species name**	**Nod**	**Fix**	**Reference**
*Listia angolensis* (Welw. ex Bak.) B.-E. van Wyk & Boatwr.	+(w)	-	[7,10]
*Listia bainesii* (Bak.) B.-E. van Wyk & Boatwr.	+	+	[7,10]
*Listia heterophylla* E. Mey.	+	+	[7,10]
*Listia marlothii* (Engl.) B.-E. van Wyk & Boatwr.	+	+	
*Listia solitudinis* (Dümmer) B.-E. van Wyk & Boatwr.	+	+	[10]
*Listia subulata* (B.-E. van Wyk) B.-E. van Wyk & Boatwr.	+	+	
*Leobordea lanata* (Thunb.) B.-E. van Wyk & Boatwr. (=*Lotononis bolusii)*	+(w)	-	[7]
*Leobordea longiflora* (H. Bolus) B.-E. van Wyk & Boatwr.	+(w)	-	[7]
*Leobordea stipulosa* (Bak. f.) B.-E. van Wyk & Boatwr.	+(w)	-	[7]
*Macroptilium atropurpureum* (DC.) Urb. cv. Siratro	+(w)	-	[10]
(w) indicates nodules present were white.			

## Genome sequencing and annotation information

### Genome project history

This organism was selected for sequencing on the basis of its environmental and agricultural relevance to issues in global carbon cycling, alternative energy production, and biogeochemical importance, and is part of the Community Sequencing Program at the U.S. Department of Energy, Joint Genome Institute (JGI) for projects of relevance to agency missions. The genome project is deposited in the Genomes OnLine Database [30] and an improved-high-quality-draft genome sequence in IMG. Sequencing, finishing and annotation were performed by the JGI. A summary of the project information is shown in Table 3.

**Table 3:** **Genome sequencing project information for *Methylobacterium *** **sp. WSM2598**

**MIGS ID**	**Property**	**Term**
MIGS-31	Finishing quality	Improved high quality draft
MIGS-28	Libraries used	Illumina GAii standard PE and CLIP PE libraries
MIGS-29	Sequencing platforms	Illumina GAii technology
MIGS-31.2	Sequencing coverage	685× Illumina
MIGS-30	Assemblers	Velvet, version 1.0.05; Allpaths r39750
MIGS-32	Gene calling method	Prodigal 1.4
	GenBank	ARAA00000000.1
	GenBank release date	August 28, 2013
	GOLD ID	Gi08887
	NCBI project ID	88639
	Database: IMG	2517572068
	Project relevance	Symbiotic N_2_ fixation, agriculture

### Growth conditions and DNA isolation

*Methylobacterium* sp. WSM2598 was grown to mid-logarithmic phase in TY rich media on a gyratory shaker at 28°C [32]. DNA was isolated from 60 mL of cells using a CTAB (Cetyl trimethyl ammonium bromide) bacterial genomic DNA isolation method [33].

### Genome sequencing and assembly

The draft genome of *Methylobacterium* sp. WSM2598 was generated at the DOE Joint Genome Institute (JGI) using Illumina technology [34,35]. For this genome, we constructed and sequenced an Illumina short-insert paired-end library with an average insert size of 270 bp which generated 19,048,548 reads and an Illumina long-insert paired-end library with an average insert size of 6354.14 +/- 3100.07 bp which generated 18,876,864 reads totaling 5,689 Mbp of Illumina data. (unpublished, Feng Chen). All general aspects of library construction and sequencing performed at the JGI can be found at the JGI website. The initial draft assembly contained 141 contigs in 41 scaffold(s). The initial draft data was assembled with Allpaths, version 39750, and the consensus was computationally shredded into 10 Kbp overlapping fake reads (shreds). The Illumina draft data was also assembled with Velvet, version 1.1.05 [36] and the consensus sequences were computationally shredded into 1.5 Kbp overlapping fake reads (shreds). The Illumina draft data was assembled again with Velvet using the shreds from the first Velvet assembly to guide the next assembly. The consensus from the second VELVET assembly was shredded into 1.5 Kbp overlapping fake reads. The fake reads from the Allpaths assembly and both Velvet assemblies and a subset of the Illumina CLIP paired-end reads were assembled using parallel phrap, version 4.24 (High Performance Software, LLC). Possible mis-assemblies were corrected with manual editing in Consed [37-39]. Gap closure was accomplished using repeat resolution software (Wei Gu, unpublished), and sequencing of bridging PCR fragments with Sanger and/or PacBio (unpublished, Cliff Han) technologies. One round of manual/wet lab finishing was also completed. 17 PCR PacBio consensus sequences were completed to close gaps and to raise the quality of the final sequence. The total (“estimated size” for the unfinished) size of the genome is 8.3 Mbp and the final assembly is based on 5,689 Mbp of Illumina draft data, which provides an average 685× coverage of the genome.

### Genome annotation

Genes were identified using Prodigal [40] as part of the DOE-JGI Annotation pipeline [41], followed by a round of manual curation using the JGI GenePRIMP pipeline [42]. Within the Integrated Microbial Genomes (IMG-ER) system [43], predicted CDSs were translated and used to search the National Center for Biotechnology Information (NCBI) nonredundant database, UniProt, TIGRFam, Pfam, PRIAM, KEGG, COG, and InterPro databases. These data sources were combined to assert a product description for each predicted protein. Non-coding genes and miscellaneous features were predicted using tRNAscan-SE [44], RNAMMer [45], Rfam [46], TMHMM [47], and SignalP [48]. Additional gene prediction analyses and functional annotation were performed within IMG.

## Genome properties

**Figure 3:**
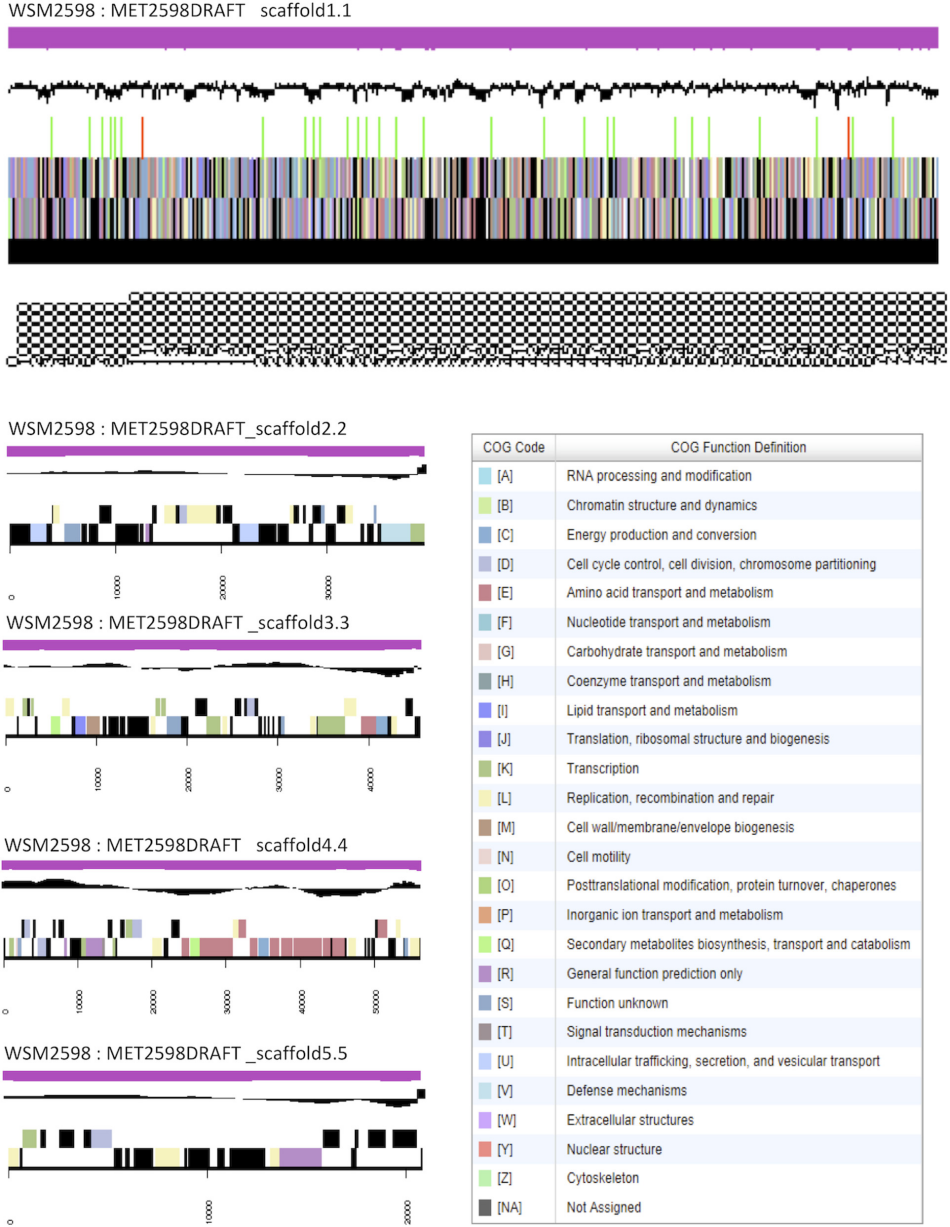
**Graphical map of the 5 scaffolds assembled for the genome of *Methylobacterium *sp. WSM2598.** From top to bottom, the scaffolds are: WSM2598: MET2598DRAFT _scaffold1.1, WSM2598: MET2598DRAFT_scaffold2.2, WSM2598: MET2598DRAFT _scaffold3.3, WSM2598: MET2598DRAFT _scaffold4.4, and WSM2598: MET2598DRAFT _scaffold5.5. From the bottom to the top of each scaffold: Genes on forward strand (color by COG categories as denoted by the IMG platform), Genes on reverse strand (color by COG categories), RNA genes (tRNAs green, sRNAs red, other RNAs black), GC content, GC skew.

The genome is 7,669,765 nucleotides with 71.17% GC content (Table 4) and comprised of 5 scaffolds (Figure 3) of 83 contigs. From a total of 7,349 genes, 7,236 were protein encoding and 18 RNA only encoding genes. The majority of genes (71.22%) were assigned a putative function whilst the remaining genes were annotated as hypothetical. The distribution of genes into COGs functional categories is presented in Table 5.

**Table 4:** **Genome statistics for *Methylobacterium *** **sp. WSM2598**

**Attribute**	**Value**	**% of total**
Genome size (bp)	7,669,765	100.00
DNA coding region (bp)	6,286,667	81.97
DNA G + C content (bp)	5,458,294	71.17
Number of scaffolds	5	
Number of contigs	83	
Total genes	7,349	100.00
RNA genes	18	0.24
rRNA operons	6	0.08
Protein-coding genes	7,236	98.46
Genes with function prediction	5,234	71.22
Genes assigned to COGs	5,025	68.38
Genes assigned Pfam domains	5,314	72.31
Genes with signal peptides	736	10.01
Genes with transmembrane helices	1,492	20.30
CRISPR repeats	3	

**Table 5:** **Number of protein coding genes of***Methylobacterium***sp. WSM2598 associated with the general COG functional categories**

**Code**	**Value**	**% age**	**COG category**
J	176	3.15	Translation, ribosomal structure and biogenesis
A	3	0.05	RNA processing and modification
K	398	7.13	Transcription
L	384	6.88	Replication, recombination and repair
B	5	0.09	Chromatin structure and dynamics
D	44	0.79	Cell cycle control, mitosis and meiosis
Y	0	0.00	Nuclear structure
V	78	1.40	Defense mechanisms
T	422	7.56	Signal transduction mechanisms
M	306	5.48	Cell wall/membrane biogenesis
N	139	2.49	Cell motility
Z	2	0.04	Cytoskeleton
W	0	0.00	Extracellular structures
U	96	1.72	Intracellular trafficking and secretion
O	155	2.78	Posttranslational modification, protein turnover, chaperones
C	399	7.15	Energy production conversion
G	307	5.50	Carbohydrate transport and metabolism
E	526	9.42	Amino acid transport metabolism
F	80	1.43	Nucleotide transport and metabolism
H	208	3.73	Coenzyme transport and metabolism
I	234	4.19	Lipid transport and metabolism
P	285	5.11	Inorganic ion transport and metabolism
Q	174	3.12	Secondary metabolite biosynthesis, transport and catabolism
R	640	11.47	General function prediction only
S	520	9.32	Function unknown
-	2,324	31.62	Not in COGS

## Conclusion

WSM2598 was sequenced as part of the DOE Joint Genome Institute GEBA-RNB project. In common with other sequenced rhizobial strains, WSM2598 has a comparatively large genome of around 7.69 Mbp, with a high proportion of genes assigned to the COG functional categories associated with transcription control and signal transduction (14.69%), transport and metabolism (29.38%) and secondary metabolite biosynthesis (3.12%). These features are characteristic of soil bacteria, which inhabit oligotrophic environments with typically diverse but scarce nutrient sources. Rhizobial methylobacteria are unusual, however, in that they form symbiotic associations exclusively with African crotalarioid legume hosts, several species of which are well-adapted to arid climates and acid, infertile soils and are therefore potentially useful pasture plants in marginal agricultural systems. The molecular basis for this symbiotic specificity has yet to be determined. As WSM2598 is highly effective for N_2_-fixation on several of these hosts, its sequenced genome is a valuable resource for gaining an understanding of symbiotic specificity and N_2_-fixation in a currently understudied group of legumes and rhizobia.

## Competing interests

The authors declare that they have no competing interests.

## Authors’ contributions

JA, JH and RY supplied the strain and background information for this project and contributed to the assembly of the manuscript with WR, TR supplied DNA to JGI and performed all imaging, WR coordinated the project and all other authors were involved in either sequencing the genome and/or editing the paper. All authors read and approved the final manuscript.

## Supplementary Material

Additional file 1: Table S1 Associated MIGS record. Click here for file
