# Supplementary material for: Genome sequence of the dark pink pigmented Listia bainesii microsymbiont Methylobacterium sp. WSM2598
Source: Stand Genomic Sci. 2014 Dec 8;9:5. doi: 10.1186/1944-3277-9-5 (PMC4334988; doi:10.1186/1944-3277-9-5)
Supplement: Additional file 1: Table S1 — Associated MIGS record. [file 1944-3277-9-5-S1.docx]

Associated MIGS Record WSM2598

**Table S1.** Associated MIGS record

| **MIGS-ID** | field name | description |
| --- | --- | --- |
| **MIGS-1** | Submit to INSDC/Trace archives |  |
| **1.1** | PID | Gi08887 |
| **1.2** | Trace Archive |  |
| **MIGS-2** | MIGS CHECK LIST TYPE |  |
| **MIGS-3** | Project Name | GEBA - Root Nodulating Bacteria |
| **MIGS-4** | Geographic Location | South Africa |
| **4.1** | Latitude | -29.032 |
| **4.2** | Longitude | 29.871 |
| **4.3** | Depth |  |
| **4.4** | Altitude | 1200 m |
| **MIGS-5** | Time of Sample collection | 2002 |
| **MIGS-6** | Habitat (EnvO) | Drakensberg Grassland biome |
| **6.1** | temperature | 28 |
| **6.2** | pH | 5-8 |
| **6.3** | salinity |  |
| **6.4** | chlorophyll |  |
| **6.5** | conductivity |  |
|  |  |  |
| **6.6** | light intensity |  |
| **6.7** | dissolved organic carbon (DOC) |  |
| **6.8** | current |  |
| **6.9** | atmospheric data |  |
| **6.10** | density |  |
| **6.11** | alkalinity |  |
| **6.12** | dissolved oxygen |  |
| **6.13** | particulate organic carbon (POC) |  |
| **6.14** | phosphate |  |
| **6.15** | nitrate |  |
| **6.16** | sulfates |  |
| **6.17** | sulfides |  |
| **6.18** | primary production |  |
| **MIGS-7** | Subspecific genetic lineage | *Methylobacterium* sp. WSM2598 |
| **MIGS-9** | Number of replicons |  |
| **MIGS-10** | Extrachromosomal elements |  |
| **MIGS-11** | Estimated Size | 7,669,765 bp |
| **MIGS-12** | Reference for biomaterial or Genome report |  |
| **MIGS-13** | Source material identifiers | WSM2598 |
| **MIGS-14** | Known Pathogenicity | Non-pathogen |
|  |  |  |
| **MIGS-15** | Biotic Relationship | Symbiotic |
| **MIGS-16** | Specific Host | *Listia bainesii* |
| **MIGS-17** | Host specificity or range (taxid) | *Listia* spp. |
| **MIGS-18** | Health status of Host | Healthy; effective nitrogen fixation |
| **MIGS-19** | Trophic Level |  |
| **MIGS-22** | Relationship to Oxygen | Aerobe |
| **MIGS-23** | Isolation and Growth conditions | TY medium[32], 28°C, aerobe |
| **MIGS-27** | Nucleic acid preparation | CTAB |
| **MIGS-28** | Library construction | Illumina GAii standard PE and CLIP PE libraries |
| **28.1** | Library size | 5,689 Mbp |
| **28.2** | Number of reads | 19,048,548 (short) 18,876,864 (long) |
| **28.3** | vector |  |
| **MIGS-29** | Sequencing method | Illumina GAii technology |
| **MIGS-30** | Assembly |  |
| **30.1** | Assembly method | Velvet, version 1.0.05, ALLPATHS r39750 |
| **30.2** | estimated error rate |  |
| **30.3** | method of calculation |  |
| **MIGS-31** | Finishing strategy |  |
| **31.1** | Status | Improved high quality draft |
| **31.2** | coverage | 685× |
| **31.3** | contigs | 83 |
| **MIGS-32** | Relevant SOPs |  |
| **MIGS-33** | Relevant e-resources |  |
